# High-resolution vessel wall imaging for quantitatively and qualitatively evaluating in-stent stenosis of intracranial aneurysms

**DOI:** 10.3389/fneur.2024.1381438

**Published:** 2024-05-09

**Authors:** Ting Chen, Shushu Liu, Yongxiang Jiang, Wei Wu, Jiali Li, Kunhua Li, Dajing Guo

**Affiliations:** ^1^Department of Radiology, The Second Affiliated Hospital of Chongqing Medical University, Chongqing, China; ^2^Department of Medical Imaging, People’s Hospital of Fengjie, Chongqing, China; ^3^Department of Neurosurgery, The Second Affiliated Hospital of Chongqing Medical University, Chongqing, China

**Keywords:** stent placement, in-stent stenosis, intracranial aneurysms, high-resolution vessel wall imaging, follow-up

## Abstract

**Background:**

It is critical to accurately and noninvasively evaluate the stented parent artery of intracranial aneurysms (IAs) with endovascular treatment.

**Objective:**

To investigate high-resolution vessel wall imaging (HR-VWI) for quantitative and qualitative evaluation of in-stent stenosis (ISS) in IAs treated with stent placement (SP).

**Methods:**

Fifty-five patients (58 aneurysms) underwent HR-VWI, contrast-enhanced (CE)-HR-VWI, CE-MR angiography (MRA), time-of-flight (TOF)-MRA, and digital subtraction angiography (DSA) six months after SP, and the reliability of quantitative stent lumen measurements was evaluated by intraclass correlation coefficient (ICC) analysis. Agreement and correlation of quantitative evaluation were estimated by comparing the four MR imaging modalities with DSA. The diagnostic performance for >0%, ≥25%, and ≥50% of ISS degrees and overall diagnostic accuracy for the ISS degrees of the four MR imaging modalities were calculated to qualitative evaluation.

**Results:**

The reliability of CE-HR-VWI and HR-VWI for ISS quantitative measurements was excellent (ICC 0.955–0.989). The agreement and correlation of CE-HR-VWI, HR-VWI versus DSA for ISS quantitative measurements were better than those of CE-MRA and TOF-MRA (*p* < 0.05). The diagnostic performance for distinguishing the degree of ISS >0%, ≥25%, and ≥50% by CE-HR-VWI and HR-VWI was superior to CE-MRA and TOF-MRA, and their overall diagnostic accuracy was 96.55 and 94.83%, respectively. HR-VWI and CE-HR-VWI were not statistically significant in the quantitative and qualitative evaluation of ISS performance (*p* > 0.05).

**Conclusion:**

HR-VWI and CE-HR-VWI have similar performance and value in the quantitative and qualitative evaluation of ISS, and HR-VWI without contrast media could be used as an ideal long-term follow-up approach after SP treatment for IAs.

## Introduction

Stent placement (SP), such as stent-assisted coil embolization or flow-diverting SP, is an important neuro-interventional endovascular treatment of intracranial aneurysms (IAs) that were previously difficult to treat with only coiling embolization (1, 2). However, SP may lead to postoperative adverse events, such as stent intima thickening and thrombosis, resulting in in-stent stenosis (ISS) or occlusion (3, 4). The incidence of ISS is relatively high, about 8.5–32.12% (3, 4), and the time of ISS occurrence varies greatly, ranging from 4 to 17 months after SP (3, 5). ISS is a dynamic process that resolves spontaneously or worsens progressively (6, 7). In addition, the stent status and degree of stenosis will affect the follow-up time, treatment plan, and prognosis of SP patients (7, 8). Thus, long-term, dynamic, and accurate evaluation of the stented parent artery is essential.

Digital subtraction angiography (DSA) is the reference standard for evaluating stent patency for SP, but it is an invasive procedure associated with complications and is not conducive to long-term follow-up of the stented parent artery. CT angiography (CTA) and contrast-enhanced (CE) MRA are noninvasive, however, stent-associated artifacts hamper the visualization and assessment of the adjacent vessel lumen, and they require the use of exogenous contrast agents, which have potentially severe side effects (9, 10). Time-of-flight (TOF)-MRA has no radiation and no contrast media, and it is easily accepted by patients, but previous study has found poor consistency between TOF-MRA with DSA (11). Compared to TOF-MRA, silent-MRA has improved the visibility of stented parent arteries, but there is insufficient evidence regarding the accuracy of detecting ISS of silent-MRA (12, 13).

High-resolution vessel wall imaging (HR-VWI) is a black blood technique based on fast spin echo sequences. Compared to other MRA sequences, HR-VWI has a smaller voxel, shorter echo time and repetition time, wider bandwidth, and parallel imaging technology (14, 15), effectively reducing the susceptibility artifact for precise configuration of the stented parent artery. Previous study revealed that HR-VWI was feasible to preliminarily evaluate the stent status (patency, stenosis), but the small sample size limited generalizability (4). Consequently, this noninvasive technique without radiation could be an ideal follow-up approach for SP. However, further quantitative and qualitative evaluation of ISS, synchronous comparison with CE-MRA and TOF-MRA, and comparison of CE-HR-VWI and HR-VWI in evaluation of ISS are rarely reported.

It was hypothesized that HR-VWI could be similar with CE-HR-VWI in quantitative and qualitative evaluation of ISS after SP treatment for IAs, and better than CE-MRA and TOF-MRA. Therefore, this study investigated CE-HR-VWI and HR-VWI for the quantitative and qualitative evaluation of ISS using DSA as the reference standard and compared its performance to CE-MRA and TOF-MRA.

## Methods

### Patients

The study was approved by our institutional review board (IRB No. 194), and written informed consent was obtained. Patients with IAs who underwent stent-assisted coil embolization or flow-diverting SP between June 2020 and February 2022 were collected. Six months after endovascular treatment, the patient was admitted for TOF-MRA, CE-MRA, HR-VWI, CE-HR-VWI, and DSA examinations. The interval between MR and DSA was less than 24 h. Patients who received double stent therapy for the same aneurysm, patients with claustrophobia and patients with incomplete or poor image quality TOF-MRA, CE-MRA, HR-VWI, CE-HR-VWI, and DSA were excluded.

### MR imaging protocol

All MR images were performed using an Ingenia CX 3.0 T MR scanner (Philips, Best, Netherlands) with a 32-channel head coil. The scanning plan was set to first scan the 3D-TOF MRA sequence for aneurysm localization and then the HR-VWI sequence of axial 3D-T1-weighted volume isotropic turbo spin echo acquisition (VISTA) for target scanning of the stented parent arteries. Then, a gadoteric acid meglumine salt injection (0.1 mmol/kg, Gd-DOTA, Jiangsu, China) was manually injected into the patient’s cubital vein for CE-MRA scanning of the head and carotid artery. The HR-VWI was repeated 5 min after the contrast agent was injected, and CE-HR-VWI images with the same range as HR-VWI were obtained. The scan parameters of the MR imaging modalities were listed in Table 1.

**Table 1:** Scan parameters of CE-MRA, TOF-MRA, and HR-VWI.

Parameters	CE-MRA	TOF-MRA	HR-VWI
FOV, mm^2^	320 × 270	200 × 181	200 × 200
Acquisition matrix	312 × 288	320 × 232	332 × 302
TR/TE, ms	3.9/1.28	21/3.45	800/22
Flip angle, °	27	18	90
Bandwidth, pix/Hz	0.412/1054	2.0/217	1.54/281.5
Slice thickness, mm	0.50	0.60	0.50
Number of slices	200	160	80
Voxel size, mm^3^	0.8 × 0.8 × 1	0.6 × 0.8 × 1.4	0.6 × 0.6 × 0.6
NEX	1	1	1
Acquisition time, min: sec	1:30	3:36	5:12

CE, contrast-enhanced; FOV, field of view; HR-VWI, high-resolution vessel wall imaging; MRA, MR angiography; NEX, number of excitations; TE, echo time; TOF, time-of-flight; TR, repetition time.

### Digital subtraction angiography

Digital subtraction angiography (DSA) was performed with an Allura X per FD 20 angiographic system (Philips, Amsterdam, Netherlands). Selective injections of the internal carotid or vertebral arteries were performed according to the aneurysm location by transfemoral catheterization. All DSA examinations included anteroposterior, lateral, and other working views. Dynamic DSA images of all views were observed, and the optimal projection angle of the target lesion was selected to measure the degree of stenosis.

### Image analysis

The four MR imaging modalities’ original thin-layer images were imported to the Philips workstation for post-processing and analysis, and multi-plane reconstruction was used to observe the original images. According to the anatomical positions of the two ends of the stent, the long and short axes of the stented parent arteries were observed in multiple planes, the image quality of the stented parent artery on the HR-VWI and CE-HR-VWI was evaluated using a 4-point scale according to the previous method (16), and the stenosis of the auxiliary stent was measured and evaluated using a submillimeter digital caliper on the MR workstation.

In the four MR imaging modalities and DSA images, the method used for determining the percentage of stenosis of a stented parent artery (immediately adjacent to or within 5 mm of the stent) was the same as that used in the Warfarin-Aspirin Symptomatic Intracranial Disease (WASID) study: percentage of stenosis = (1 − [D_stenosis_/D_normal_]) × 100, where D_stenosis_ is the diameter of the stented parent artery at the site of the most severe stenosis and D_normal_ is the diameter of the proximal lumen of the normal stented parent artery adjacent to the stenosis (17, 18). When there was no change in the parent artery diameter, cases were graded as no stenosis (4); otherwise, cases were graded as intimal hyperplasia (1–24%), mild (25–49%), moderate (50–74%), or severe (≥75%) (19).

The four MR imaging modalities and DSA images were independently reviewed by two neuroradiologists (both with >10 years of experience). When a different reading was proposed in evaluating the image quality of the four MR imaging modalities, the two radiologists reached a consensus after an in-depth discussion. The four MR imaging modalities and DSA were measured separately without knowledge of the four MR imaging modalities or DSA examination results. The location of the aneurysms to be evaluated was provided to the readers, and all measurements were performed twice by two observers. To reduce possible memory effects, the measurements were performed with an interval of four weeks between the readings.

### Statistical analysis

All statistical analyses were performed using SPSS 26.0 software (IBM SPSS Inc., Chicago, IL, United States). Quantitative variables were described as mean ± SD or median (interquartile range), and qualitative variables were described as numbers and percentages. Intra- and inter-observer reliability of ISS quantitative measurements in the five imaging modalities were assessed by calculating the intraclass correlation coefficient (ICC). The agreement and correlation of the four MR imaging modalities versus DSA were compared with ICCs, Bland–Altman plots, and scatter plots. Sensitivity, specificity, positive predictive value (PPV), negative predictive value (NPV), area under the curve (AUC) value for diagnoses of >0%, ≥25%, and ≥50% stenosis, and overall diagnostic accuracy for the different degrees of ISS were calculated. The consistency of the four MR imaging modalities and DSA in the degree of stenosis >0%, ≥25%, and ≥50% and overall different degrees of ISS were evaluated using *κ* statistics. The Delong test was used to pairwise compare the AUC of the receiver operating characteristic (ROC) curve for the four MR imaging modalities for ISS of >0%, ≥25%, and ≥50%. The color software package was used to pairwise compare the ICC values of reliability of the Intra- and inter-observers, the ICC values of agreement with DSA, and the *r* values of correlation with DSA in the four MR imaging modalities for ISS measurements (20). A *p*-value <0.05 was considered statistically significant. ICC, *κ*, *r*, and AUC values were, respectively, interpreted as ICC values <0.50 (poor), 0.50–0.75 (moderate), 0.75–0.90 (good), and >0.90 (excellent), *κ* values<0.40 (poor), 0.40–0.54 (weak), 0.55–0.69 (moderate), 0.70–0.84 (good) and 0.85–1.00 (excellent), *r* values<0.10 (negligible), 0.10–0.39 (weak), 0.40–0.69 (moderate), 0.70–0.89 (strong) and ≥0.90 (very strong), AUC values <0.5 (chance), 0.51–0.59 (very poor), 0.60–0.69 (poor), 0.70–0.79 (moderate), 0.80–0.89 (good) and ≥0.90 (excellent) (21, 22).

## Results

### Patient characteristics

**Figure 1:**
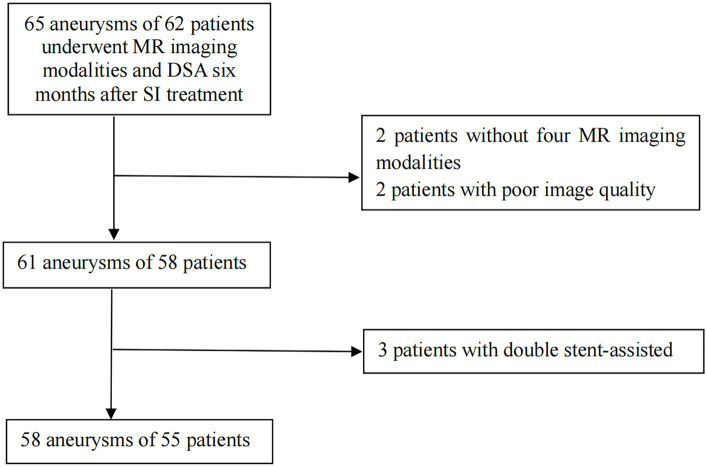
Flowchart of patients selection. DSA, digital subtraction angiography; SI, stent implantation.

The flowchart of patients selection is shown in Figure 1. In total, 58 aneurysms of 55 patients were collected in this study, and the patients were readmitted after 6.96 ± 1.46 months. The patients’ characteristics are summarized in Table 2. The four types of stents involved in this study were LVIS stents (Microvention, Tustin, CA, United States) used for 39 aneurysms, Neuroform Atlas stents (Stryker Neurovascular, California, United States) used for 11 aneurysms, Pipeline stents (Coviden/ev3 Neurovascular, Irvine, CA, United States) for used for 4 aneurysms and Tubridge stents (MicroPort Neuro-Tech, Shanghai, China) used for 4 aneurysms, respectively. The Flow-diversion (Pipeline and Tubridge) ISS was 25.00%(2/8) and the stent (LVIS and Neuroform Atlas) ISS was 36.00%(18/50).

**Table 2:** Characteristics of the patients and aneurysms.

Characteristics	Value
Mean age^a^	54.17 ± 9.15
Female/male	43/15 (74.14%/25.86%)
Hypertension	32 (55.17%)
Diabetes	4 (6.90%)
Coronary heart disease	3 (5.17%)
Hyperlipidemia	27 (46.55%)
Smoking history	12 (20.69%)
Ruptured aneurysms	42 (72.41%)
Multiple intracranial aneurysms	3 (5.17%)
Original size of the aneurysm (mm)^a^	4.33 ± 2.08
Location of the aneurysm Internal carotid artery^b^ Anterior communicating artery Basilar artery Middle cerebral artery Bifurcation Parietal	37 (63.79%) 12 (20.69%) 5 (8.62%) 4 (6.89%) 32 (55.17%) 26 (44.83%)
Operation situation No complications Stent thrombosis Intraoperative cerebral hemorrhage	56 (96.55%) 1 (1.72%) 1 (1.72%)
Degrees of ISS	
Intimal hyperplasia (1–24%)	16.00% ± 5.22%
Mild (25–49%)	34.00% ± 7.22%
Moderate (50–74%)	58.05% ± 4.44%

a Mean ± standard deviation.

b Including carotid terminus and origins of the posterior communicating and ophthalmic artery.

### Image quality of CE-HR-VWI and HR-VWI

The average image quality scores of CE-HR-VWI and HR-VWI were 3.88 ± 0.33 and 3.86 ± 0.35, respectively, and not significantly different.

### Intra- and inter-observer reliability for ISS quantitative measurements

For ISS measurement, there was excellent intra- and inter-observer reliability for CE-HR-VWI (ICC 0.968–0.987), HR-VWI (ICC 0.955–0.989), and DSA (ICC 0.978–0.994) but only good reliability for CE-MRA and TOF-MRA (ICC 0.839–0.893), except excellent inter-observer reliability for CE-MRA (ICC, 0.932).

### Agreement and correlation of the four MR imaging modalities versus DSA for ISS quantitative measurements

The ICC analysis demonstrated excellent agreement between CE-HR-VWI and HR-VWI with DSA for ISS measurement (ICC 0.994, 0.992, respectively) but a poor agreement between CE-MRA and TOF-MRA with DSA (ICC 0.281, 0.187, respectively).

**Figure 2:**
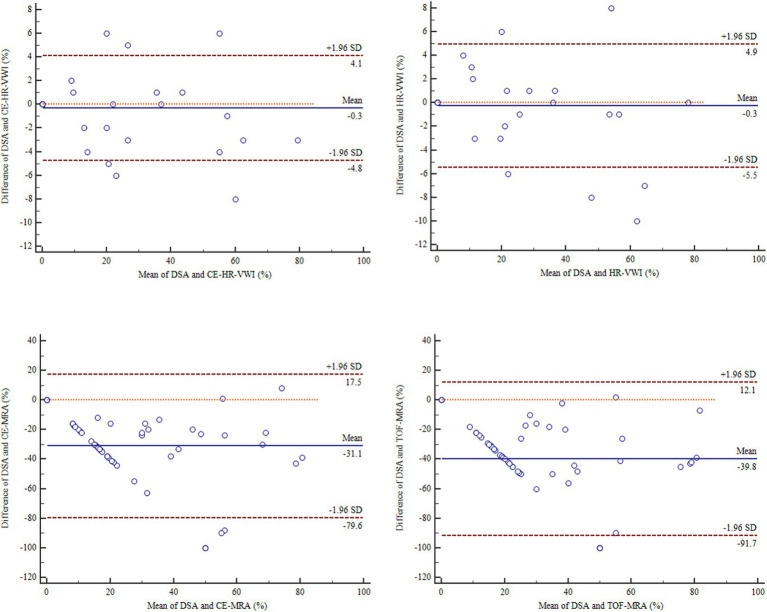
Bland-Altman plots results of the agreement between the four MR imaging modalities and DSA in the ISS quantitative measurements. CE, contrast-enhanced; DSA, digital subtraction angiography; HR-VWI, high-resolution vessel wall imaging; MRA, MR angiography; TOF, time-of-flight.

Bland-Altman plots depicted that CE-HR-VWI and HR-VWI exhibited good agreement with DSA in ISS measurements [mean bias both −0.3, 95% limits of agreement (−4.8–4.1%), (−5.5–4.9%), respectively], whereas CE-MRA and TOF-MRA displayed poor agreement with DSA [mean bias = −31.1, −39.8%, respectively, 95% limits of agreement (−79.6–17.5%) (−91.7–12.1%), respectively] (Figure 2).

**Figure 3:**
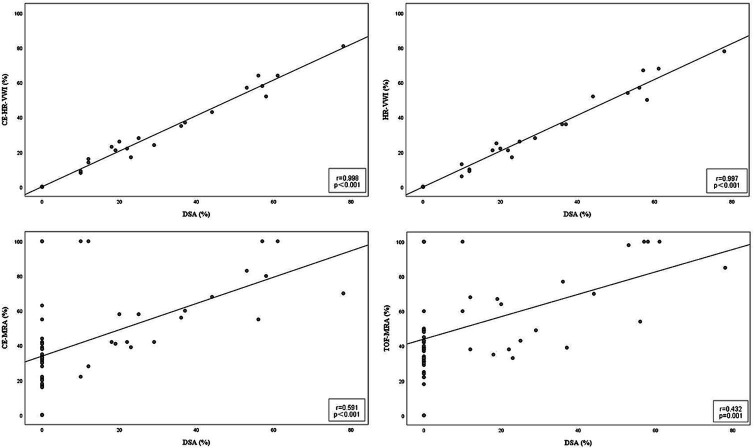
Scatter plots results of the correlation between the four MR imaging modalities and DSA in the ISS quantitative measurements. CE, contrast-enhanced; DSA, digital subtraction angiography; HR-VWI, high-resolution vessel wall imaging; MRA, MR angiography; TOF, time-of-flight.

As illustrated in Figure 3, CE-HR-VWI and HR-VWI strongly correlated with DSA in the ISS measurements (*r* 0.998, 0.997, respectively), while CE-MRA and TOF-MRA moderately correlated with DSA (*r* 0.591, 0.432, respectively).

### Accuracy of qualitative evaluation for ISS among the four MR imaging modalities

The diagnostic performance is presented in Table 3. The AUC values for distinguishing >0%, ≥25%, and ≥50% degrees of stenosis of CE-HR-VWI and HR-VWI were excellent diagnostic performance (AUC 99.7–100%). The AUC values for distinguishing >0%, ≥25%, and ≥50% degrees of stenosis of CE-MRA demonstrated good diagnostic performance (AUC 83.8–89.9%). The AUC for distinguishing ≥50% stenosis of TOF-MRA depicted good diagnostic performance (AUC 87.7%) but distinguishing the degree of ISS >0% and ≥25% demonstrated moderate diagnostic performance (AUC 74.4 and 79.4%, respectively). CE-HR-VWI and HR-VWI demonstrated good to excellent agreement with DSA in identifying the degree of ISS >0%, ≥25%, and ≥50% (*κ* 81.4–100%), while CE-MRA and TOF-MRA showed poor agreement with DSA (*κ* 5.6–23.4%) (Table 3). One of ≥50% ISS cases was ≥75% ISS. CE-HR-VWI, HR-VWI, and TOF-MRA all distinguished such stenosis, but TOF-MRA significantly overestimated the degree of stenosis.

**Table 3:** The diagnostic performance for >0%, ≥25%, and ≥50% ISS of the four imaging modalities using DSA as a reference standard.

	>0%	≥25%	≥50%
CE-HR-VWI analysis (*n* = 58)			
Sensitivity, % (95%CI)	100.0 (80.0–100.0)	90.9 (57.1–99.5)	100.0 (51.7–100.0)
Specificity, % (95%CI)	100.0 (88.6–100.0)	97.9 (87.3–99.9)	100.0 (91.4–100.0)
PPV, %(95%CI)	100.0 (80.0–100.0)	90.9 (57.1–99.5)	100.0 (51.7–100.0)
NPV, % (95%CI)	100.0 (88.6–100.0)	97.9 (87.3–99.9)	100.0 (91.4–100.0)
AUC, % (95%CI)	100.0 (100.0–100.0)	99.8 (99.2–100.0)	100.0 (100.0–100.0)
*κ* value, %(95%CI)	100.0 (100.0–100.0)	88.8 (73.5–104.1)	100.0 (100.0–100.0)
HR-VWI analysis (*n* = 58)			
Sensitivity, % (95%CI)	100.0 (80.0–100.0)	100 (67.9–100.0)	83.3 (36.5–99.1)
Specificity, % (95%CI)	100.0 (88.6–100.0)	97.9 (87.3–99.9)	98.1 (88.4–99.9)
PPV, % (95%CI)	100.0 (80.0–100.0)	91.7 (59.8–99.6)	83.3 (36.5–99.1)
NPV, % (95%CI)	100.0 (88.6–100.0)	100.0 (90.4–100.0)	98.1 (88.4–99.9)
AUC, % (95%CI)	100.0 (100.0–100.0)	100.0 (100.0–100.0)	99.7 (98.7–100)
*κ* value, % (95%CI)	100.0 (100.0–100.0)	94.6 (84.0–105.2)	81.4 (56.3–106.5)
CE-MRA analysis (*n* = 58)			
Sensitivity, % (95%CI)	100.0 (80.0–100.0)	100.0 (67.9–100.0)	100.0 (51.7–100.0)
Specificity, % (95%CI)	15.8 (6.6–31.9)	31.9 (19.5–47.3)	76.9 (62.8–87.0)
PPV, % (95%CI)	38.5 (25.6–53.0)	25.6 (14.0–41.5)	33.3 (14.4–58.8)
NPV, % (95%CI)	100.0 (51.7–100.0)	100.0 (74.7–100.0)	100.0 (89.1–100.0)
AUC, % (95%CI)	83.8 (72.9–94.6)	87.9 (79.2–96.6)	89.9 (81.5–98.3)
*κ* value, % (95%CI)	11.5 (1.9–21.1)	15.1 (4.1–25.5)	23.4 (6.5–40.3)
TOF-MRA analysis (*n* = 58)			
Sensitivity, % (95%CI)	100.0 (80.0–100.0)	100.0 (67.9–100.0)	100.0 (51.7–100.0)
Specificity, % (95%CI)	7.9 (2.1–22.5)	14.9 (6.7–28.9)	71.2 (56.7–82.5)
PPV, % (95%CI)	36.4 (24.1–50.5)	21.6 (11.8–35.7)	28.6(12.2–52.3)
NPV, % (95%CI)	100.0 (31.0–100.0)	100.0 (56.1–100.0)	100.0 (88.3–100.0)
AUC, % (95%CI)	74.4 (61.6–87.2)	79.4 (67.5–91.3)	87.7 (78.1–97.2)
κ value, % (95%CI)	5.6 (−0.9–12.1)	6.2 (0.5–11.8)	17.1 (3.8–30.4)

AUC, area under the curve; CE, contrast-enhanced; CI, confidence interval; HR-VWI, high-resolution vessel wall imaging; MRA, MR angiography; NLR, negative likelihood ratio; NPV, negative predictive value; PLR, positive likelihood ratio; PPV, positive predictive value; TOF, time-of-flight.

In the distinguishing >0%, ≥25%, and ≥50% stenosis, the AUC values of the four MR imaging modalities were significantly different in the pairwise comparison, except for CE-HR-VWI versus HR-VWI, which was not significantly different.

**Figure 4:**
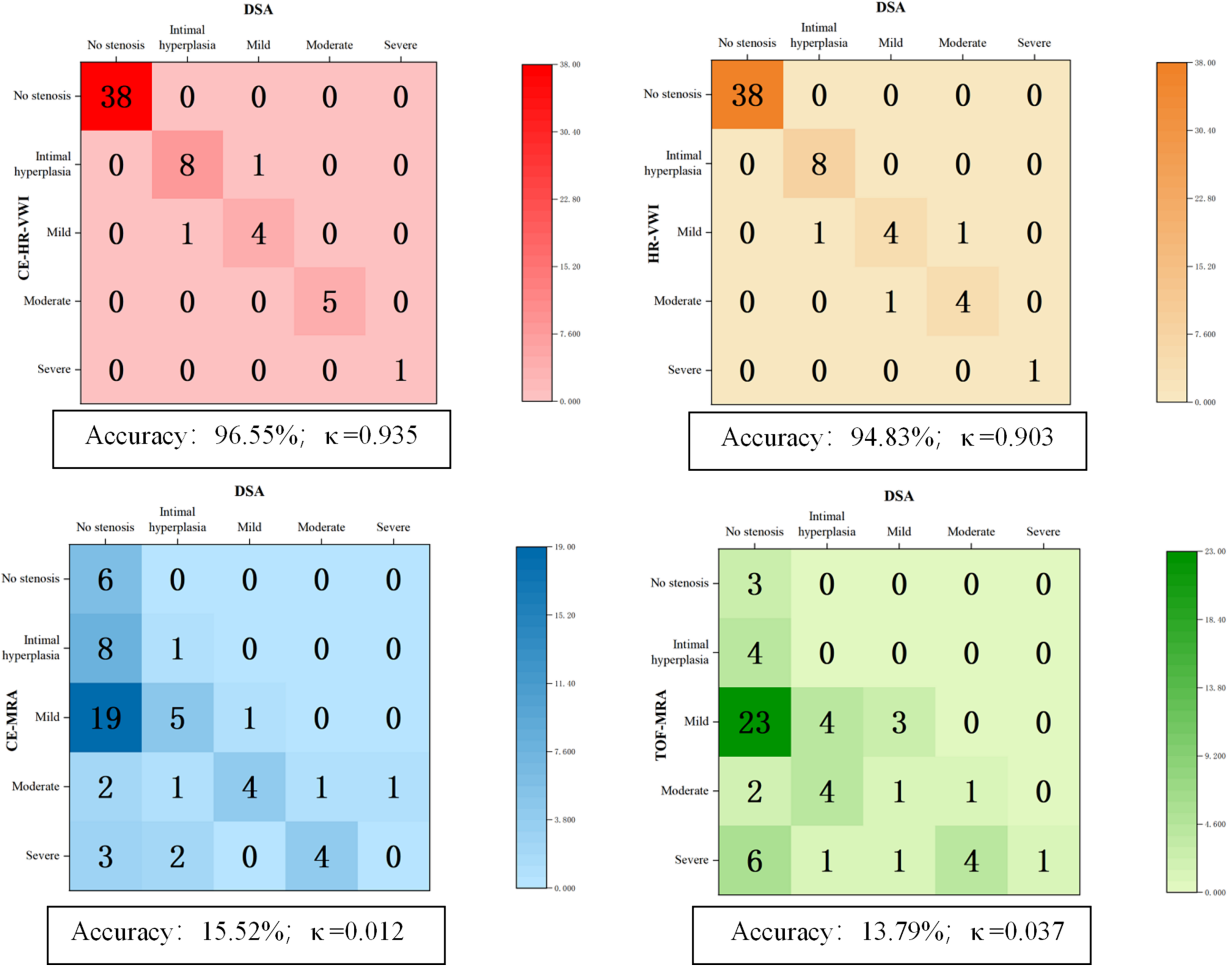
The overall consistency and accuracy for the different degrees of ISS of the four MR imaging modalities using DSA as the standard. CE, contrast-enhanced; DSA, digital subtraction angiography; HR-VWI, high-resolution vessel wall imaging; MRA, MR angiography; TOF, time-of-flight.

**Figure 5:**
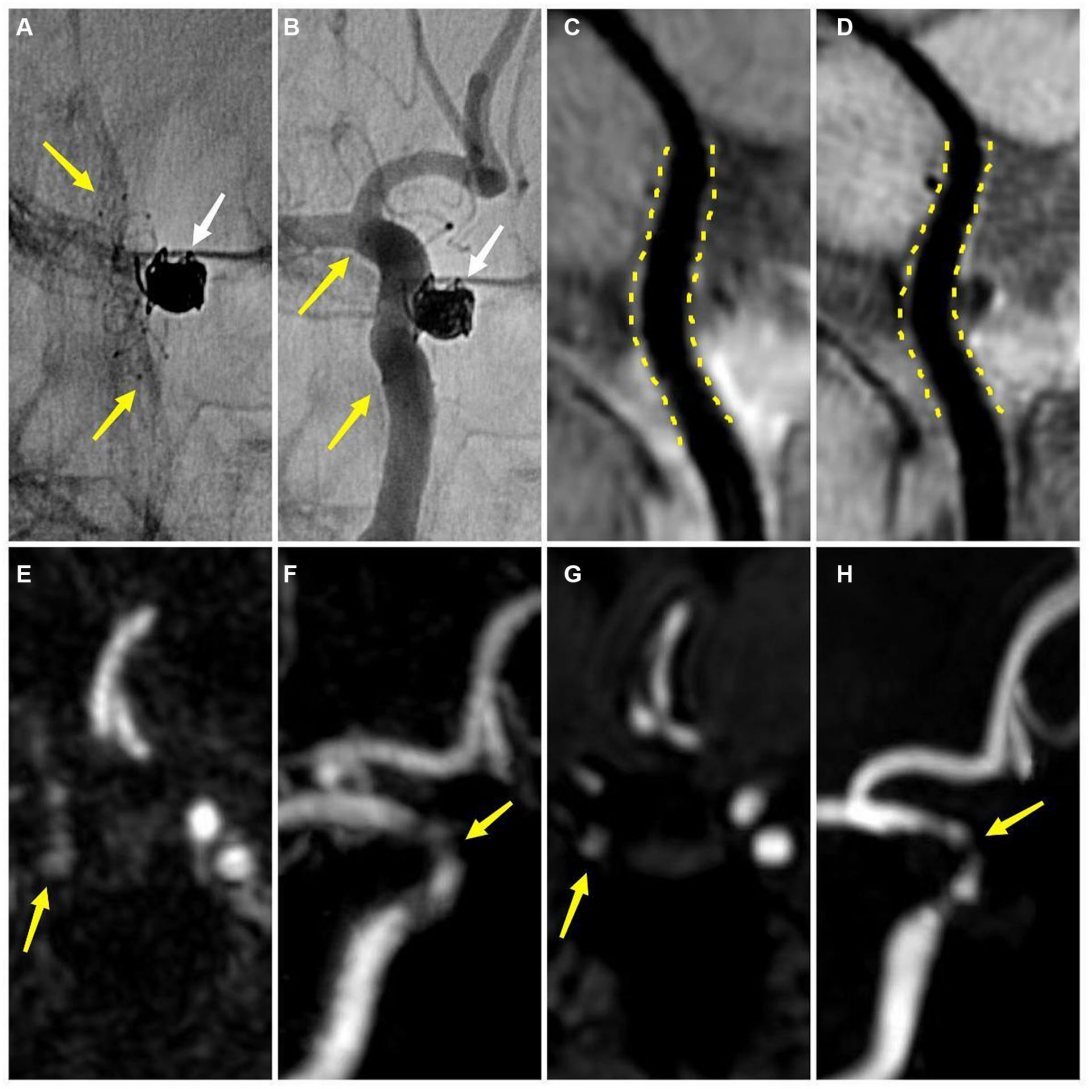
An adult patient with a left internal carotid artery aneurysm after stent-assisted coil embolization about 6 months **(A,B)** DSA demonstrates the embolized aneurysm (white arrow) and the LVIS stent (yellow arrow) **(B)** DSA shows no ISS **(C)** CE-HR-VWI and **(D)** HR-VWI reveal no ISS (yellow dotted line) **(E,F)** CE-MRA shows that the degree of ISS is 28% (yellow arrow) **(G,H)** TOF-MRA demonstrates that the degree of ISS is 42% (yellow arrow). DSA, digital subtraction angiography; ISS, in-stent stenosis; CE, contrast enhanced; HR-VWI, high-resolution vessel wall imaging; MRA, MR angiography; TOF, time-of-flight.

**Figure 6:**
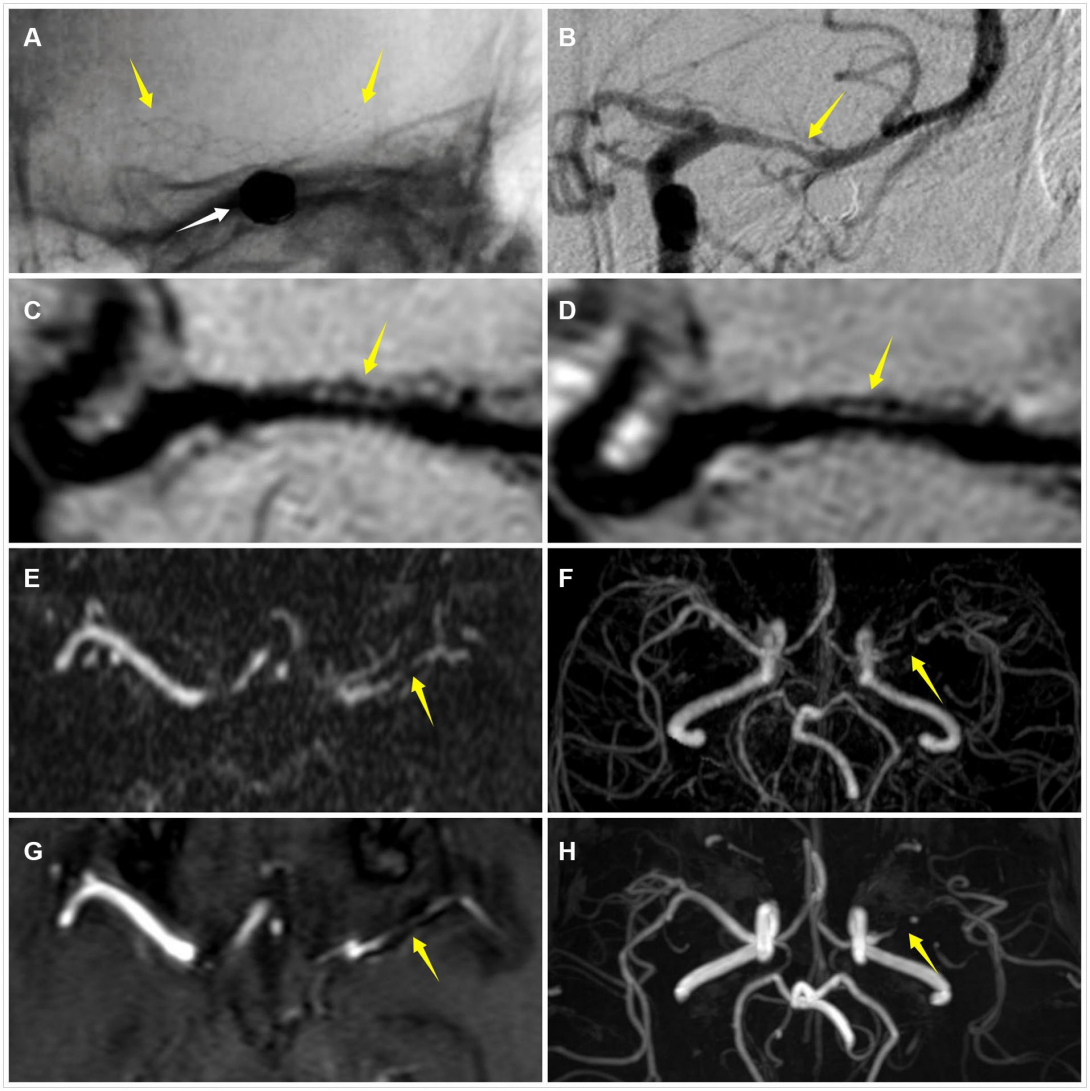
An adult patient with a left middle cerebral artery aneurysm after stent-assisted coil embolization about 8 months **(A)** DSA demonstrates the embolized aneurysm (white arrow) and the LVIS stent (yellow arrow) **(B)** DSA shows that the degree of ISS is 58% (yellow arrow) **(C)** CE-HR-VWI reveals that the degree of ISS is 61% (yellow arrow) **(D)** HR-VWI demonstrates that the degree of ISS is 64% (yellow arrow) **(E,F)** CE-MRA and **(G,H)** TOF-MRA show that the degrees of ISS are almost 100% (yellow arrow). DSA, digital subtraction angiography; ISS, in-stent stenosis; CE, contrast enhanced; HR-VWI, high-resolution vessel wall imaging; MRA, MR angiography; TOF, time-of-flight.

Using DSA as the reference standard, the overall diagnostic accuracy of CE-HR-VWI, HR-VWI, CE-MRA, and TOF-MRA in the different degrees of ISS was 96.55, 94.83, 15.52, and 13.79%, respectively. CE-HR-VWI and HR-VWI had excellent consistency with DSA in the overall identification of different degrees of ISS (*κ* 0.935 and 0.903, respectively), while CE-MRA and TOF-MRA had poor consistency with DSA (*κ* 0.012 and 0.037, respectively) (Figures 4–6).

## Discussion

To our knowledge, this is the first study to investigate HR-VWI and CE-HR-VWI for the quantitative and qualitative evaluation of ISS after SP treatment for IAs using DSA as the reference standard and synchronously compared its performance to CE-MRA and TOF-MRA. And we found that HR-VWI and CE-HR-VWI performed significantly better than CE-MRA and TOF-MRA for the quantitative and qualitative evaluation of ISS, while the performance of HR-VWI and CE-HR-VWI was very similar.

Precise quantitative measurement of the lumen is a prerequisite for accurate qualitative evaluation of the stented parent artery, which depends on measurement reliability and accuracy. The reliability of measurement is mainly reflected in the repeatability and reproducibility, and in the present study, the repeatability (intra-observers) and reproducibility (inter-observers) of CE-HR-VWI and HR-VWI in all ISS measurements were almost perfect and superior to CE-MRA and TOF-MRA. This may be closely related to the excellent image quality and features of HR-VWI, which can suppress the flow signals and stent artifacts and acquire precise configuration of the stented parent artery lumen and wall. Furthermore, the black blood background in the lumen of the HR-VWI sequence can help observers to identify the boundary of the lumen of the stented parent artery and the abnormal signal lesions on the stent wall (15), which is suitable for repeated multiple measurements of the stented parent arteries.

Regarding the accuracy of stent lumen quantitative measurement, we found that CE-HR-VWI and HR-VWI had an excellent agreement with DSA and a strong correlation with DSA. The Bland–Altman plots results demonstrated very small bias, relatively narrow 95% confidence intervals, and small measured data dispersion between CE-HR-VWI and HR-VWI versus DSA. Besides, the ICC values of CE-HR-VWI and HR-VWI versus DSA were up to 0.992 to 0.994, which indicated that the ISS measurements of them were very similar. While in our study, 32 (32/58, 55.17%) cases of CE-MRA and 35 (35/58, 60.34%) cases of TOF-MRA misjudged no stenosis to different degrees of stenosis in scatter plots (Figure 5), and there were large data dispersion, and bias in the scatter plots and Bland–Altman plots of CE-MRA and TOF-MRA. This may be because the loss and deformation of signal caused by stent metal artifacts led to overestimation of ISS (Figure 6) (10, 11), therefore, CE-HR-VWI and HR-VWI were superior to CE-MRA and TOF-MRA for the quantitative evaluation of ISS.

Accurately qualitative evaluation of the degree of ISS is very important for clinical management and follow-up of SP. The stent of stenosis <25% is expected and beneficial to achieve occlusion of the aneurysm, which requires only routine follow-up (19, 23), and 25–49% ISS has the potential for more severe stenosis or occlusion (5, 7), while ≥50% ISS is prone to TIA or cerebral infarction, so requires more intensive follow-up, drug control or endovascular therapy (5, 7). Previous studies have found that HR-VWI was feasible in assessing the status of the stent (stenosis or patency) after SP for IAs (4, 24, 25). Compared to TOF-MRA, HR-VWI had higher accuracy for evaluating stented parent arteries and excellent agreement with DSA (*κ* = 1) (4). In our study, CE-HR-VWI and HR-VWI displayed excellent diagnostic accuracy and good to excellent agreement with DSA in qualitative evaluation of the degree of ISS >0%, ≥25%, and ≥50%, which were better than CE-MRA and TOF-MRA. Regarding the diagnostic performance, the sensitivities of the four MRI modalities were very high, while the specificity, PPV, and PLR values of CE-HR-VWI and HR-VWI were significantly higher than those of CE-MRA and TOF-MRA. Marciano et al. (10) and Akkaya et al. (11) also found that CE-MRA and TOF-MRA had poor overall consistency with DSA in ISS degrees. These results implied that CE-HR-VWI and HR-VWI were superior to CE-MRA and TOF-MRA in identifying ISS, possibly because CE-MRA and TOF-MRA are sensitive to stent and coil metal artifacts and failed to accurately quantitative measure the lumen of stented parent arteries (4, 10).

Accurate interpretation of HR-VWI findings in clinical practice requires considering the conditions of adequate flow suppression, optimal spatial resolution, and reasons for enhancement after administration of a gadolinium-containing contrast agent. Previous studies have reported that CE-HR-VWI may show vessel wall enhancement artifacts and result in pseudo vascular wall thickening lesions (26).The image quality of CE-HR-VWI and HR-VWI were excellent and not significantly different in our study. CE-HR-VWI and HR-VWI had similar repeatability and reproducibility in-stent lumen measurement and diagnostic accuracy and consistency with DSA in ISS measurements and degrees. Therefore, the unenhanced HR-VWI might be preferable to assess ISS alone in clinical practice since it avoids the potential side effects of gadolinium toxicity and nephrogenic systemic fibrosis (27, 28). However, CE-HR-VWI might be needed if further exploration of the cause of ISS is required, such as additional information on the enhancement of stented parent arteries wall or lumen (18).

The current study has some limitations. First, this study was a single-center design, and the total sample size and the number of cases of ISS were relatively small. Second, since only one case was severe ISS (≥75%), we did not specifically analyze the diagnostic efficacy of severe ISS. Third, the sample size was insufficient for comparing different stent types and sizes. Fourth, for the HR-VWI technology, we only reported the comparison between the 3D T1-VISTA sequence and DSA, so other HR-VWI sequences versus DSA need to be validated in the future.

## Conclusion

HR-VWI and CE-HR-VWI demonstrated excellent diagnostic accuracy and consistency with DSA in the quantitative and qualitative evaluation of ISS after SP treatment for IAs and was superior to CE-MRA and TOF-MRA, therefore as a noninvasive and effective imaging modality, HR-VWI without contrast media may be an alternative to DSA and ideal follow-up approach for detecting ISS.

## Data availability statement

The raw data supporting the conclusions of this article will be made available by the authors, without undue reservation.

## Ethics statement

The studies involving humans were approved by Ethics Committee of the Second Hospital of Chongqing Medical University. The studies were conducted in accordance with the local legislation and institutional requirements. The participants provided their written informed consent to participate in this study. Written informed consent was obtained from the individual(s) for the publication of any potentially identifiable images or data included in this article.

## Author contributions

TC: Data curation, Methodology, Writing – original draft. SL: Data curation, Formal analysis, Software, Writing – review & editing. YJ: Data curation, Project administration, Writing – review & editing. WW: Methodology, Software, Validation, Writing – review & editing. JL: Data curation, Formal analysis, Writing – review & editing. KL: Conceptualization, Writing – review & editing. DG: Funding acquisition, Project administration, Supervision, Writing – review & editing.
